# Comparing forms of neighborhood instability as predictors of violence in Richmond, VA

**DOI:** 10.1371/journal.pone.0273718

**Published:** 2022-09-06

**Authors:** Samuel J. West, Diane Bishop, Derek A. Chapman, Nicholas D. Thomson

**Affiliations:** 1 Department of Psychology, Virginia State University, Petersburg, Virginia, United States of America; 2 Department of Surgery, Virginia Commonwealth University, Petersburg, Virginia, United States of America; 3 Division of Epidemiology, Department of Family Medicine & Population Health, Virginia Commonwealth University, Petersburg, Virginia, United States of America; Utrecht University: Universiteit Utrecht, NETHERLANDS

## Abstract

Violence events tend to cluster together geospatially. Various features of communities and their residents have been highlighted as explanations for such clustering in the literature. One reliable correlate of violence is neighborhood instability. Research on neighborhood instability indicates that such instability can be measured as property tax delinquency, yet no known work has contrasted external and internal sources of instability in predicting neighborhood violence. To this end we collected data on violence events, company and personal property tax delinquency, population density, race, income, food stamps, and alcohol outlets for each of Richmond, Virginia’s 148 neighborhoods. We constructed and compared ordinary least-squares (OLS) to geographically weighted regression (GWR) models before constructing a final algorithm-selected GWR model. Our results indicated that the tax delinquency of company-owned properties (e.g., rental homes, apartments) was the only variable in our model (R^2^ = 0.62) that was associated with violence in all but four Richmond neighborhoods. We replicated this analysis using violence data from a later point in time which yielded largely identical results. These findings indicate that external sources of neighborhood instability may be more important to predicting violence than internal sources. Our results further provide support for social disorganization theory and point to opportunities to expand this framework.

## Introduction

Violence is a major public health problem in the United States. Rates of homicide in the US are 7.50 times that of other high-income nations [1]. This problem is more pronounced in Richmond, VA, where the leading cause of death among young people is violence [2]. Epidemiological research has placed an emphasis on the environments in which violence is most likely to occur, revealing that waves of violence occur similarly to viral epidemics, spreading like a contagious disease [3]. A clear picture emerges from this research: low-income and otherwise underserved communities are at the highest risk for being exposed to violence in some way [4]. In the current work we drew from social disorganization theory to examine the impact of various geospatial features on neighborhood-level violence data from Richmond, VA.

### Theoretical accounts of violence

Several theoretical models have been put forward in the literature to explain observed trends in violence and antisocial behavior. Social disorganization theory (SDT) places a major emphasis on specific elements of the environment a person resides in explaining such antisocial behavior [5,6]. SDT broadly identifies important physical features of communities such as physical disorder (e.g., dilapidated buildings) [7] and meaningful social ties (e.g., with one’s neighbors) [8] as primary factors in predicting antisocial behaviors. Evidence indicates that stronger social ties in a community facilitate a sort of *collective efficacy* via expanded support networks and other forms of resource sharing which may provide access to a broader array of mediation options when conflicts may arise [9,10]. This element is also theorized to ameliorate the negative effects of societal impacts originating from outside one’s own neighborhood (e.g., poverty; residential instability) [11]. In support of these assertions research indicates that various forms of violence (e.g., intimate partner violence, homicide) are directly linked with poor collective efficacy and low stability (i.e., social disorganization) in a given neighborhood [12,13]. These findings have been replicated in urban and rural communities [14] and other countries such as Brazil and South Africa [15,16]. One major source of such disorganization identified by SDT is that of residential instability.

### Neighborhood residential stability and violence

Neighborhood residential instability refers to how likely it is for members of a given neighborhood to relocate, typically due to the loss of one’s home. At a conceptual level low neighborhood stability makes good sense as a predictor of violence. Consistent with SDT, neighborhoods with high resident turnover are likely to have poor social bonds among neighbors which can lead to little willingness to work together with others towards common goals (i.e., reducing or preventing violence) [17]. Recent work further indicates that low neighborhood stability is a strong predictor of interpersonal violence [18,19]. Various operational definitions of neighborhood instability have been applied in the literature. Some work has utilized measures of tenure or the proportion of the residents who recently moved into a given neighborhood. However, this measure may be too general to identify the probable sources of such residential turnover which may account for the weak relationships observed between measures of tenure and rates of crime [20]. Given the emphasis SDT places on sources of disorganization (i.e., endogenous versus exogenous disorganization) distinguishing between such sources of residential instability may further our understanding of the influence of this factor on violence.

Another measure that has emerged as appropriate for examining social disorganization at the neighborhood level is that of property tax delinquency [21]. The number of tax delinquent properties in a given residential area captures crucial structural differences across neighborhoods which are strongly linked with greater socioeconomic disadvantage, poorer community health, and greater neighborhood disorganization and distress [21,22]. Underscoring these differences, those from high tax delinquency neighborhoods are also more likely to die at an earlier age [22]. Similarly, a recent study found that the number of tax delinquent and vacant properties in a neighborhood also predict greater rates of violent crime [23]. Despite this evidence, no known research has examined the potential for divergent sources of neighborhood instability to differentially predict violence at the neighborhood level.

### Contrasting sources of instability: Company and personal tax delinquency

In general, there are two forms of tax delinquency that comprise most delinquent residential properties: those owned by companies or landlords (i.e., rental properties) and those owned by the residents themselves. The implications of personal tax delinquency are relatively clear when considered in the context of SDT: owners of run-down homes are less likely to keep up the financial state of their home much like the physical state of their home, thus introducing social disorder and contributing to an environment that is conducive to violence [24]. SDT also indicates that such physical disorder is likely to be intervened upon by other residents as neighborhoods are endogenously self-stabilizing [25]. However, the implications of tax delinquent company-owned properties are less clear. In the case of rental properties, the tenants themselves often have little say in the state of the building they live in and are typically at the mercy of their landlord for repairs or improvements, which may diminish the ability of collective efficacy to address such physical disorder. Tax delinquent rental properties hold unique implications for the housing security of tenants, as many localities have laws allowing properties that have been in delinquency for a given period to be foreclosed and auctioned to other investors. Such transactions are directly linked to eviction rates and thus pose a critical threat to the collective efficacy of a community in turn [26].

For example, in the city of Richmond, VA properties that are tax delinquent for two years are commonly seized by the city and auctioned to recoup lost tax revenues. Richmond (like many other localities) has no official regulation protecting tenants in the case that winners of auctioned delinquent properties decide to evict them. Evidence indicates that real estate companies frequently purchase such delinquent properties, targeting low-income neighborhoods and communities of color, ultimately disrupting any resident-based revitalization or improvement efforts [27]. The effects of company property tax delinquency may thus be distinct from private tax delinquency as company tax delinquency appears to contribute to both physical disorder and residential instability and may thus poses a greater threat to collective efficacy. Although there is no extant empirical literature comparing the impact of company and personal tax delinquency directly, one example revealing the impact of company delinquency does exist.

The East Liberty neighborhood had one of the highest crime rates in Philadelphia in 2008. A group of residents decided to form a real-estate investment group, named East Liberty Development Inc. (ELDI), for the purpose of revitalizing many of the dilapidated, tax delinquent properties in their neighborhood in hopes of reducing crime rates [28]. After numerous interviews with East Liberty residents, ELDI leadership concluded that there was a connection between crime rates and slumlords–that is, rental property owners who had not taken care of their rental properties despite having tenants. ELDI thus began its “slumlord buyout” program, wherein they purchased run down rental properties from slumlords in order to renovate them in collaboration with the current tenants. Studies examining the impact of this program revealed that violent crimes (i.e., aggravated assault, sexual assault, homicide) decreased by 49% over a four-year period [29,30]. As such, it appears that rental properties with absentee landlords (e.g., those holding tax delinquent properties) may predict violence in a given neighborhood, but it remains unclear if this variable is a more appropriate predictor of violence than the tax delinquency of personal properties. A proper investigation of these relationships must account for the geospatial clustering commonly observed with violence.

### Geospatial features of violence

Geospatial analyses have been used in various fields (e.g., epidemiology) to determine patterns and predictors of violence in selected geographical regions. This approach has yielded meaningful insights that can be applied towards effective localized interventions. For example, applying geospatial analysis to violence data can reveal high-risk neighborhoods that can be targeted with intervention efforts [4]. Indeed, severe violence events are most likely to occur within the victim’s own neighborhood [31]. Those living in densely populated, low-income, and racially diverse neighborhoods are at a significantly higher risk of violence than those from predominantly affluent, white neighborhoods [32]. One analytic technique known as Geographically Weighted Regression (GWR) [33] accounts for the spatial heterogeneity of data by allowing the estimated parameters to vary across each designated region while accounting for the influence of neighboring regions. This approach allows researchers to examine the extent to which predictor variables account for a given outcome (i.e., violence) in each region (e.g., neighborhoods) and at the global level (e.g., a whole city). For example, research applying this approach indicates that the density of alcohol outlets predicts levels of violence [32,34]. Population density has also repeatedly emerged as an important geospatial predictor of violence [35]. Other work using GWR indicates that food insecurity is a strong predictor of gun violence [36]. Research reveals that cities historically affected by racist ‘redlining’ housing practices demonstrate spatial heterogeneity in statistical models predicting forms of antisocial behavior due to the protracted effects of the discriminatory policies used to shape the demographics of affected neighborhoods [37–39].

### Current study

Geospatial analyses have revealed much about spatial patterns of violence and its predictors. Neighborhood instability (operationalized here as property tax delinquency) is a known predictor of various outcomes including rates of violence at the neighborhood level. However, no known work has examined the potential for different forms of tax delinquency (i.e., sources of instability) to yield distinct impacts on violence. To address this gap in the literature we collected data from the Richmond City, VA on violent crimes and both forms of tax delinquency. We also collected data to control for known predictors of violence: population density, neighborhood race, alcohol dispensing outlets, median income, and food insecurity. We estimated and compared Ordinary Least Squares (OLS) regression and GWR models to geospatially explore violence in Richmond neighborhoods. The current work is exploratory in nature and thus we did not make any predictions *a priori*. All data and code used in our analyses are publicly available on the Open Science Framework (https://osf.io/fsx6z/files).

## Methods

### Ethics statement

All data collected for the current investigation comprised publicly available archival data and thus no ethics approval was sought for these variables. However, our final violence index variable did contain data obtained from records from Virginia Commonwealth University Health’s emergency department. Consent was waived (i.e., not directly obtained) for the collection of these data and the protocol utilized was given ethics review and approval by the institutional review board at Virginia Commonwealth University (protocol: HM13846).

### Data acquisition

#### Tax delinquency

Tax delinquency data were obtained from the City of Richmond’s Open Data repository website (https://data.richmondgov.com/). The tax delinquency reports generated by the City of Richmond contain information regarding the owner of the property, the amount in delinquency, the address of the property, and the geospatial coordinates (i.e., longitude and latitude) for each property. Properties in the original dataset comprised those that were in delinquent tax status for six months or more at the time of our initial query (6/30/2021). We then created two variables on the basis of the type of property: those owned by companies and those owned by private individuals prior to preprocessing.

#### Neighborhood regions

Richmond neighborhoods were determined based on the city’s publicly available neighborhood dataset and shapefile (https://data.richmondgov.com/). At the time of this study the city of Richmond officially recognized 148 neighborhoods.

#### Neighborhood characteristics

We obtained estimates of neighborhood demographics from the Statistical Atlas, an online tool that provides neighborhood level estimates based on data from the US Census Bureau and the American Communities Survey (https://statisticalatlas.com). We measured population density as the number of persons per square mile. Neighborhood demographics were characterized by median income, racial makeup (as the proportion of white residents), and the proportion of residents receiving food stamps. We also counted the number of active alcohol dispensing outlets existed within each neighborhood using the public database of currently active ABC licenses maintained by the city of Richmond (https://data.richmondgov.com/Economic-Growth/City-of-Richmond-ABC-Licenses/bc34-rjtz).

#### Violence

We obtained data on neighborhood violence from two sources. First, we pulled the reported crime rates from the public database that is maintained by the Richmond Police Department (RPD; https://apps.richmondgov.com/applications/CrimeInfo). We collected count data on the number of homicides and assaults reported from January 1^st^, 2021 until June 30^th^, 2021. We then summed the number of homicides and assaults to create a single violence events variable. Second, we obtained data on violent crimes through the internal patient information database for violence-related visits to VCU Health’s emergency department (ED) within the same timeframe among patients aged 10–24. These data were acquired via an existing research protocol from the VCU Clark Hill Institute for Positive Youth Development. We also pulled data from the RPD database on violence events that occurred after this initial data collection period (i.e., July 1^st^–October 31^st^) in order to examine a direct replication of our model and to ensure that any observed effects were not due to the time of year during which our initial data was collected as the seasonality of violence is well known [40].

### Analytic approach

#### Data pre-processing

To create our separate tax delinquency variables, we first removed all duplicate entries from the raw datafile. This was necessary because the City of Richmond appends each new monthly report of delinquent tax properties to the end of the prior month’s report–we retained all the most-recently reported properties. We then manually assessed each property in the resultant dataset to determine if the property belonged to a company or an individual. This was done by inspecting the ownership information included in the City of Richmond’s data. Properties that belonged to organizations that were distinctly different from companies (i.e., religious organizations) were excluded. Similarly, locations that did not have a clear owner indicated or were marked as “Unknown Owner” were also removed. We additionally removed two large sections of delinquent properties (42 addresses total) that comprised undeveloped wooded areas and belonged to two companies. However, we did include vacant lots that existed within neighborhoods as unkempt vacant lots in urban areas show similar effects on violence and other outcomes to blighted properties with residential structures [41]. Our final company tax delinquency variable comprised information on 586 properties, whereas our final personal tax delinquency variable comprised 1,837 properties. These data were then loaded into R statistical software version 4.1.0 [42]. We used the *st_intersects* function of the sf package to count the number of each type of delinquent properties contained within each neighborhood as defined by the boundaries from the City of Richmond’s neighborhoods shapefile [43].

#### Primary analyses

We used the *lm* function from base R to conduct our OLS regressions [42]. We tested the spatial dependency of our variables and OLS residuals using the *moran*.*test* and *lm*.*morantest* functions from the spdep package, respectively [44]. We also used the *poly2nb* and *nb2listw* functions from spdep to identify neighboring regions and assign spatial weights, respectively. For our initial GWR models we determined the optimal adaptive bandwidths with gaussian kernels using the *gwr*.*sel* function and used the *gwr* function from the spgwr package to estimate our initial models [45]. The model fit of our initial OLS and GWR models was compared using the *LMZ*.*F2GWR*.*test* function from spgwr. For our final GWR model we applied the least absolute shrinkage and selection operator (LASSO) to arrive at the most parsimonious model possible by using the *gwl*.*est* function from the gwrr package [46]. The LASSO accomplishes this by applying a machine learning algorithm that penalizes model coefficients (i.e., reduces the estimate to zero) for each identified region (i.e., neighborhoods) and then re-examines the model fit until it can no longer be improved, a process known as regularization [47]. This procedure is used to address multicollinearity (i.e., between our tax delinquency variables) and to control for model complexity. LASSO-identified models thus do not rely on *p-*values to determine the significance of a given predictor variable. It is instead assumed that if the LASSO includes a non-zero parameter estimate that parameter improves the model fit and is thus interpreted similarly to an OLS regression coefficient with *p* < .05. Given that our analyses and GWR models generally are considered to be exploratory, the LASSO was an ideal approach allowing us to balance model precision with possible discovery. Finally, all visualizations were created using the tmap package [48].

## Results

### Preliminary analyses

**Fig 1:**
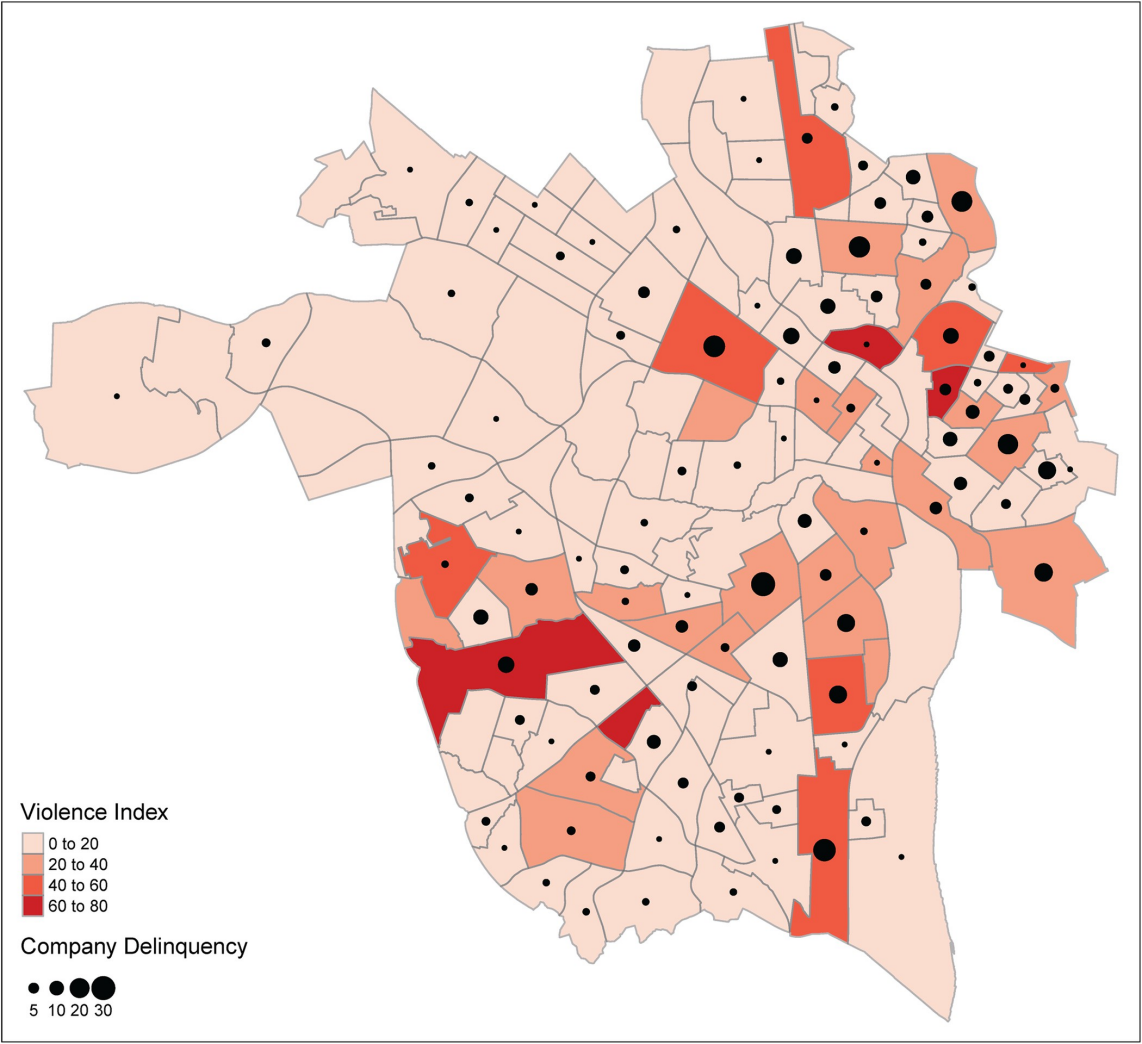
Geospatial distribution of the violence index in relation to company tax delinquency.

Descriptive statistics and zero-order bivariate correlations of all study variables are available in Tables 1 and 2, respectively. We tested the spatial dependence of our variables as a data lacking spatial dependency are not appropriate for GWR. The RPD-obtained violence variable yielded a significant Moran’s *I*, indicating that these events were indeed spatially dependent, *I* = 0.11, *SD* = 2.25, *p* = .012. We found similar results for our ED-obtained data on violence events, such that the data demonstrated significant spatial dependence, *I* = 0.10, *SD* = 2.07, *p* = .019. These variables were also strongly correlated with one another, *r*(146) = 0.56, *p* < .001. Given this degree of similarity, we summed these variables to create a single violence index for each neighborhood. We then examined the spatial dependency of our predictor variables. Population density showed significant spatial dependence, *I* = 0.24, *p* < .001, as did race, *I* = 0.72, *p* < .001, median income, *I* = 0.67, *p* < .001, alcohol outlets, *I* = 0.19, *p* < .001, food stamps, *I* = .61, *p* < .001, company-owned tax delinquent properties, *I* = 0.14, *p* = .002, and personal tax delinquent properties, *I* = 0.12, *p* = .008. The spatial distribution of violence and company delinquent properties is displayed in Fig 1.

**Table 1:** Descriptive statistics for all study variables.

Variables	*M*	*SD*	Min	Max
**Alcohol Outlets**	2.30	6.86	0.00	60.00
**Company Delinquency**	3.94	5.73	0.00	30.00
**Food Stamps**	0.19	0.16	0.00	0.81
**Median Income**	47085.78	32086.63	0.00	231750.00
**Personal Delinquency**	12.25	15.43	0.00	76.00
**Population Density**	4275.51	3809.82	0.00	20355.00
**Proportion White**	0.36	0.31	0.00	0.98
**Violence Index**	12.80	15.25	0.00	72.00

**Table 2:** Zero-order bivariate correlations among all study variables.

		1	2	3	4	5	6	7
**1**	**Alcohol Outlets**	-						
**2**	**Company Delinquency**	.21***	-					
**3**	**Food Stamps**	-.23**	.20*	-				
**4**	**Income**	.02	-.23**	-.55***	-			
**5**	**Population Density**	.23**	.19*	.20*	-.27**	-		
**6**	**Proportion White**	.27***	-.22**	-.66***	.68***	-.08	-	
**7**	**Private Delinquency**	-.03	.80***	.30***	-.23**	.17*	-.26**	-
**8**	**Violence Index**	.20*	.45***	.45***	-.38***	.43***	-.32***	.43***

Notes.

*p < .05

**p < .01

***p < .001.

### OLS models

Before estimating our initial GWR models we first estimated simplified OLS regressions for each of our tax delinquency variables, controlling for population density, to compare against their GWR counterparts. In OLS Model 1, we modeled the violence index as our outcome predicted by population density and the company tax delinquency. This model accounted for 32% of the overall variance in the violence index, where tax delinquent properties, *β* = 0.39, *p* < .001, and population density, *β* = 0.36, *p* < .001 were both significant positive predictors of violence. OLS Model 2 was identical except we replaced the company tax delinquency variable with private tax delinquency. This model accounted for 31% of the variability in violence and again revealed significant effects of tax delinquent properties, *β* = 0.37, *p* < .001, and population density, *β* = 0.37, *p* < .001. We then applied the Moran’s test examine the spatial autocorrelation of the residuals from each model. These tests revealed that the residuals from OLS Model 1, Moran’s *I* = .08, *p* = .037, and OLS Model 2, Moran’s *I* = .11, *p* = .015, both demonstrated significant spatial heterogeneity, suggesting that a spatial regression model was appropriate. Given that our outcome variable was a count variable in nature, we re-conducted these regression analyses using a negative binomial distribution. Our results from these additional analyses were largely identical to those from our OLS regressions (Supplemental Document 1).

### GWR models

We re-estimated our OLS models as GWR models which accounted for spatial heterogeneity among the data at the neighborhood level. GWR Model 1 implemented population density and company tax delinquency as predictors of our violence index. The ideal adaptive bandwidth was determined to be 0.24. GWR Model 1 accounted for 41% of the variability in the violence index and a generally improved model fit, *AICc* = 1166.27, relative to OLS Model 1, *AICc* = 1174.94. We also compared the model fit of these models statistically. This analysis revealed that GWR Model 1 was indeed a significantly better fit to the data, *F*(14.65, 145) = 2.41, *p* = .004. The residuals from GWR Model 1 also no longer demonstrated significant spatial dependence, indicating that it was properly accounted for by the GWR, *I* = 0.02, *p* = 0.284. We then estimated GWR Model 2 using personal tax delinquency as our main predictor and compared it to OLS Model 2. The ideal adaptive bandwidth was determined to be 0.56. GWR Model 2 accounted for approximately 34% of the variability in the violence index, and a similar model fit, *AICc* = 1175.66, in relation to OLS Model 1, *AICc* = 1177.41. Similarly, our model comparison failed to indicate that GWR Model 2 was a significant improvement over OLS Model 2, *F*(9.03, 145) = 1.85, *p* = .063. Likewise, some spatial heterogeneity remained from GWR Model 2, *I* = .09, *p* = .036.

### Final GWR model: Applying the LASSO

Although the private delinquency GWR model was not better than the OLS model, the Moran’s test statistic indicated spatial dependency of this variable and the spatial dependency found among the residuals from OLS Model 2. This suggested that it was important for us to control for this predictor in our final model. Proper estimation of a GWR model however must address multicollinearity among the explanatory variables as GWR is sensitive to multicollinearity [49]. In our case, some of our predictors were strongly correlated (e.g., the tax delinquency variables), requiring some correction in our model estimation in order to directly compare the impact of the sources of neighborhood instability. We addressed this issue by using the LASSO to penalize our local coefficients in order to select the best fitting model to our data while addressing this collinearity. We also added several important control variables to our final model. Specifically, the proportion of white neighborhood residents, median income, number of alcohol outlets, and the proportion of residents receiving food stamps to our model as control variable because each of these components have emerged as predictors of neighborhood violence in past work.

**Fig 2:**
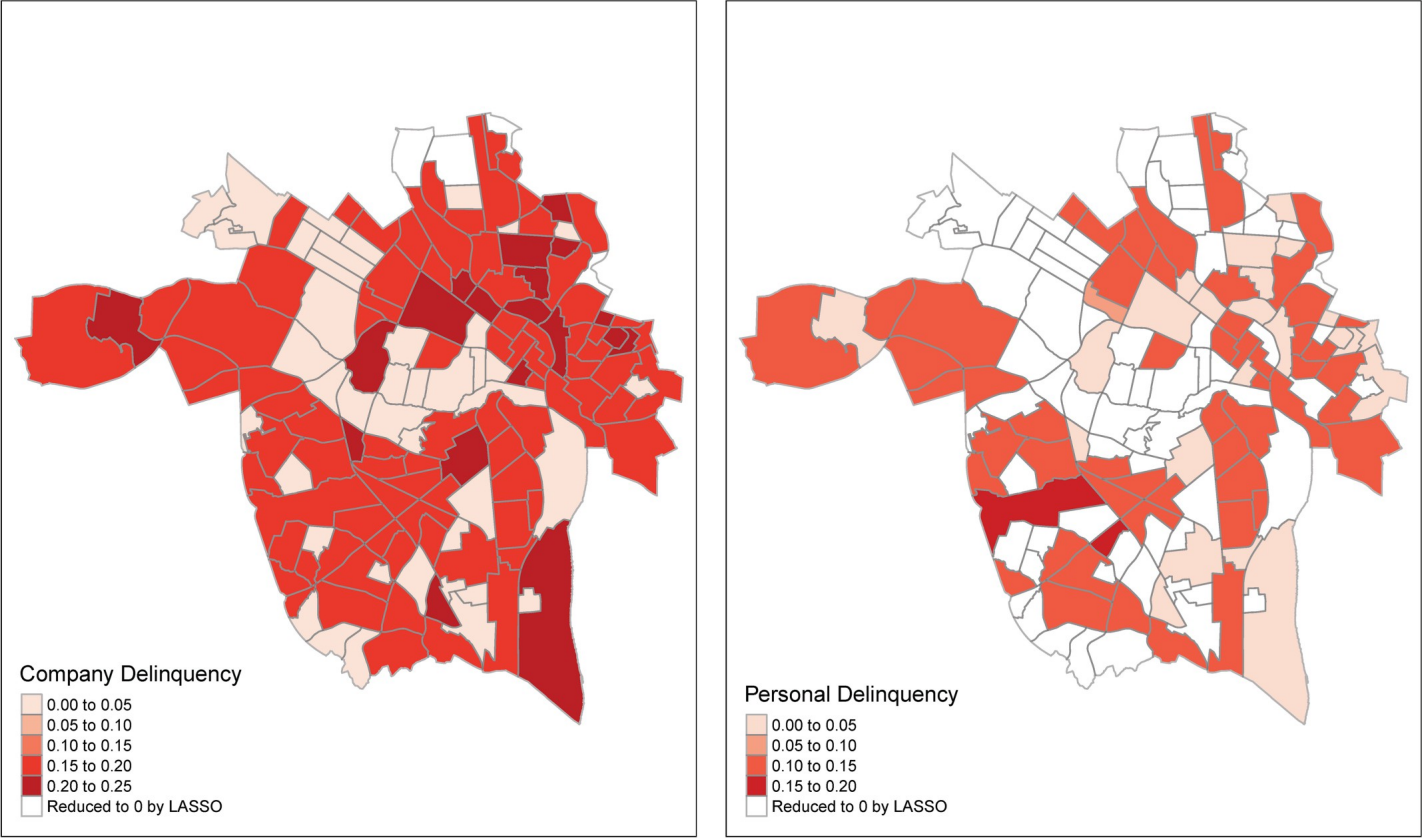
Geospatial distribution of standardized beta coefficients for company (left) and personal (right) delinquency.

The final model identified by the LASSO accounted for 62% of the variance in the violence index overall. Inspection of the neighborhood-level coefficients indicated that of predictors included in our model company tax delinquency (Fig 2) and food stamps were retained as predictors of violence in most of the 148 Richmond neighborhoods (excepting 4 and 19 neighborhoods, respectively), whereas population density, personal tax delinquency, proportion of white residents, median income, alcohol outlets, and food stamps yielded more 0 coefficient estimates (45, 68, 135, 75, and 92 respectively). These findings generally indicate that the LASSO found that variance from the violence index was best accounted for in some neighborhoods when uniquely attributed to these predictors rather than all of them. Similarly, company tax delinquency exhibited greater relative importance than personal delinquency at the global level (Table 3). Inspection of the model residuals indicated that no spatial dependence remained, *I* = .02, *p* = .298.

**Table 3:** Standardized estimates of variables in the final LASSO GWR model.

Variable	Min.	1^st^ Quantile	2^nd^ Quantile	3^rd^ Quantile	Max.	*M*	*SD*
**Population Density**	0.00	0.00	0.23	0.30	0.34	0.19	0.13
**Company Delinquency**	0.00	0.03	0.17	0.18	0.20	0.13	0.08
**Personal Delinquency**	0.00	0.00	0.01	0.12	0.16	0.05	0.06
**Proportion White**	-0.03	0.00	0.00	0.00	0.00	-0.00	0.01
**Median Income**	-0.07	-0.06	0.00	0.00	0.00	-0.02	0.03
**Alcohol Outlets**	0.00	0.00	0.00	0.15	0.20	0.06	0.08
**Food Stamps**	0.00	0.03	0.20	0.31	0.33	0.18	0.12

We also estimated two simultaneous spatial autoregression models to compare the relative strengths of our tax delinquency variables in a different analytic framework that still allowed us to account for spatial heterogeneity. These analyses revealed that company and private tax delinquency demonstrate similar associations with violence with company tax delinquency evincing marginally stronger relations at the global and direct impact levels when modeled separately (S1-S4 Tables in S1 File). A relative importance analysis also revealed that company tax delinquency accounted for more variance in violence events than did private delinquency across every level of model complexity (i.e., predictors included in the model; S5 Table in S1 File).

### Examining errors

**Fig 3:**
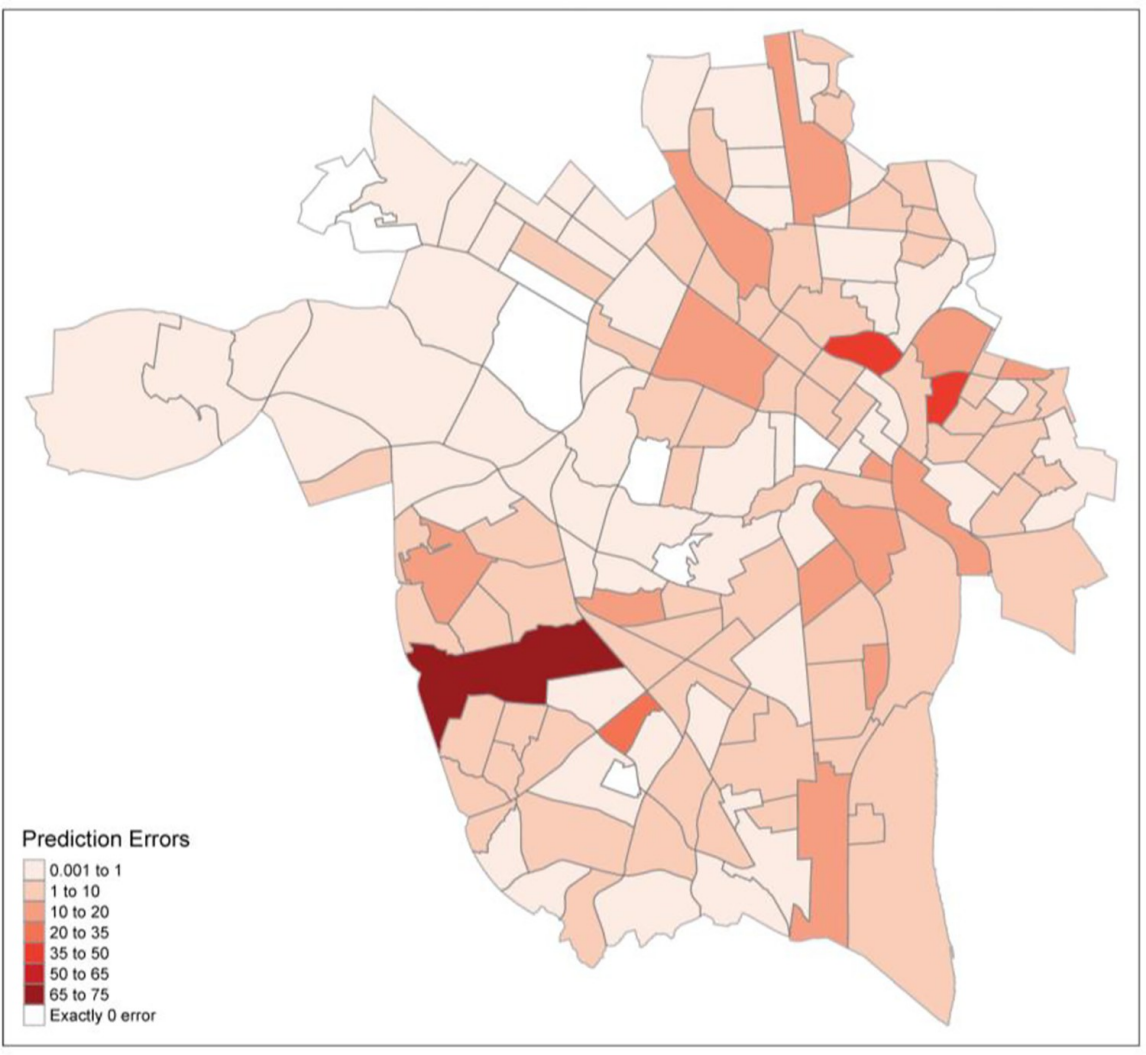
Geospatial distribution of model prediction errors.

In order to identify the neighborhoods that our model did not fit well we created a general error index by finding the absolute value of our model residuals. Error index values closer to zero reflected the most accurate predictions made by our model. Given that our error index was in raw units (i.e., differences between count values) it gave us a clear indication of how many erroneous violence events were predicted in each neighborhood. Examination of these values revealed that our model yielded reasonable accuracy in many areas of Richmond (Fig 3). Specifically, 65 neighborhoods had an error index value of less than 1, indicating that predictions about these neighborhoods were the most accurate of our model. One clear theme that emerged among the neighborhoods with exactly zero errors (*n* = 9) was that all these areas also had zero violence events. Our model performed quite well in neighborhoods with higher violence rates as well. For example, the North Highland Park neighborhood had a relatively high violence index (33) and very little prediction error, as our model predicted 32.49 violence events in this neighborhood. In contrast, our model performed poorly in some neighborhoods. Inspection of the high error neighborhoods revealed two trends. First, several of the high error neighborhoods contained the Richmond City housing courts which are comprised primarily of subsidized public housing administered by the Richmond Redevelopment and Housing Authority (e.g., Gilpin Court, Mosby Court, Whitcomb Court). Second, other areas within these high-error neighborhoods were largely commercial (e.g., Midlothian).

### Replication model

The replication violence data was strongly correlated with the combined violence index used in our initial analyses, *r*(146) = .87, *p* < .001. We thus preceded conduct a direct replication of our combined model with LASSO selection using violence data from a different point in time as our outcome variable. The resultant model explained 76.66% of the violence that occurred in these months. Inspection of the global model coefficients further revealed similar results to our initial LASSO such that company tax delinquency emerged as a more important predictor than personal delinquency in predicting violence (Table 4). The replication model yielded 88 neighborhoods with error values less than 1.

**Table 4:** Standardized estimates of variables in the replication LASSO GWR model.

Predictor	Minimum	1st Quantile	2^nd^ Quantile	3rd Quantile	Maximum	*M*	*SD*
**Population Density**	0.00	0.05	0.21	0.23	0.26	0.15	0.09
**Company Delinquency**	0.06	0.11	0.28	0.30	0.38	0.22	0.14
**Personal Delinquency**	-0.08	0.00	0.00	0.00	0.00	-0.01	0.02
**Proportion White**	0.00	0.00	0.00	0.00	0.02	0.00	0.00
**Median Income**	-0.13	-0.10	-0.08	0.00	0.00	-0.05	0.05
**Alcohol Outlets**	0.00	0.00	0.26	0.32	0.33	0.17	0.14
**Food Stamps**	0.00	0.00	0.15	0.20	0.25	0.10	0.10

## Discussion

Violence spreads like a contagious disease. As a result, violence events tend to be clustered together geospatially in regions that are characterized by social disorganization. But role might the *source* of such disorganization play in such community violence? In the current work we applied LASSO-regularized geographically weighted regression (GWR) to violence data from Richmond, VA’s 148 neighborhoods to compare two sources of social disorganization via neighborhood instability (i.e., company vs. personal property tax delinquency) as predictors of violence at the neighborhood level. Our results indicated that violence events in Richmond, VA were indeed spatially dependent and that company tax delinquency, rather than personal, was a stronger predictor of violence in most neighborhoods. As such, it appears that external sources of neighborhood instability may play an important role in predicting violence in such communities.

### Geospatial features of violence and neighborhood instability

First, our analyses revealed that violence events in Richmond, VA are indeed spatially dependent. Specifically, high-violence neighborhoods tended to be located close to other high-violence neighborhoods, suggesting that a collection of geospatial features may explain the violence within these specific regions. This finding is consistent with myriad literature evincing such spatial dependence among violence events [50,51]. Next, we found that population density positively predicted violence in most neighborhoods. This finding is also consistent with past research and makes good sense–the more densely packed people are into a given environment the more likely conflict is to arise [52]. We also found that the company tax delinquency variable was the most consistent predictor of violence in our model as it was the only predictor variable with a non-zero parameter estimate almost every neighborhood. This finding is broadly consistent with the reported violence-reducing effects of slumlord buyout programs [28,29]. Our findings further suggested that personal tax delinquency does not meaningfully predict violence in most neighborhoods after accounting for company tax delinquency and our other control variables. Taken together, this evidence suggests that the neighborhood instability caused by those outside a given community (e.g., negligent landlords and business owners), rather than residents, may play a significant role in fostering an environment conducive to violence in cities like Richmond, VA.

### Implications for theories of violence and anti-social behavior

Our finding that company delinquency was generally a more important predictor of personal delinquency is consistent with a social disorganization account of violence. Specifically, SDT indicates that sources of disorganization that pose direct threats to the collective efficacy of a community are likely to play a larger role in predicting negative outcomes. Company tax delinquency may be more important as a predictor of violence than personal delinquency for this reason. That is, personal delinquency may be directly addressed by the collective intervention of other community members, whereas individual community members are often powerless against company tax delinquency and the associated negative effects (i.e., residential instability). Although our findings are generally consistent with this account, our data and analyses are unable to provide evidence that company tax delinquency directly impacts collective efficacy and thus more research is needed in this domain.

In this view it is not the residents of high-violence neighborhoods that have constructed a disorganized environment conducive to antisocial behaviors, but those who hold power over the structural features of these neighborhoods, despite not residing within them. Our findings are also generally consistent with work indicating that the transmission of violence occurs similarly to a viral infection at the population level [3]. Indeed, the high violence neighborhoods identified in the current work all directly bordered other high violence neighborhoods with one exception. Strikingly, our plot of these data made clear that the northwest region of Richmond, VA had very low levels of violence and property tax delinquency. We also observed a small section of neighborhoods in Richmond’s central southside that exhibited elevated levels of instability yet had little violence despite being bordered by higher violence neighborhoods on all sides. The lack of violence in this region is even more striking as Richmond’s southside is generally comprised of underserved neighborhoods in relation to the affluent upper west end. This finding is consistent with the surveillance data published by the VCU Clark Hill Institute which indicates that these regions were stable in their rates of violence from 2008 to 2019 [53]. Finally, our violence index and replication violence variables were strongly correlated to the point of redundancy, indicating that violence events were generally occurring in the same places over time.

### Implications for public policy

Although the current findings cannot directly uncover the causal mechanisms between company tax delinquency and violence, they do point to some concrete changes that may ameliorate such factors influencing violence. Our results suggest that so-called ‘slumlords’ (i.e., rental property owners that do not maintain their rental properties) may play a role in the violence seen in Richmond’s neighborhoods. Previous research indicates that the purchase of tax delinquent properties by larger real estate firms in economically depressed areas is a major contributor to inequality and thus to violence [27]. As such, it would seem one method to bolster neighborhood stability and thus reduce violence would be for localities to develop regulations barring the purchasers of auctioned tax delinquent properties from evicting the current tenants without due cause. One simple solution to this could be to require the purchasers of delinquent rental properties to uphold any outstanding lease agreement between the current tenants and prior owner.

### Limitations

Despite the strength of our findings, they must be considered within the context of several limitations. First, this project acquired the majority of our data from publicly available data repositories and thus we were unable to verify the quality of the data collected. Specifically, the data collected from RPD indicate the number of calls investigated for a given reason rather than a set of confirmed crimes whereas the data collected from the VCU ED comprised only information about adolescents (10–24 years old). Future work should examine whether these effects are consistent across age groups. Second, because our analyses relied on the neighborhood boundaries defined by the city of Richmond, we were limited to using data that was already linked to neighborhoods. As a result, the demographic data used in our models (i.e., population density and race) were based on data collected by the American Communities Survey which was completed 2012–2016 and thus our models are based on older estimates of these variables. We relied on these estimates because the violence data available from RPD is only available at the aggregate, neighborhood level and more recent estimates of these variables were only available at the census tract level. Further, the RPD violence data comprised the majority of violence events codified in our dependent variable and thus was important to retain in our analysis. Third, some regions identified as neighborhoods by the city of Richmond are distinctly not neighborhoods. For example, Belle and Mayo Islands comprise one of the neighborhoods in Richmond, yet these are two small islands in the James river that hold public parks, but no residential buildings. As a result, our models included values of zero for the proportion of white residents in these areas. We retained these regions in our analyses because they had non-zero values for the violence index, and we sought to include the entire city in our analyses. Similarly, several neighborhoods in Richmond are made up of universities and contain much student housing (e.g., VCU). The drastically different demographics found in such neighborhoods (i.e., age) may impact the relationships examined in the current investigation, but our data did not allow us to examine such possibilities. Future work is needed to examine the generalizability of our findings in this context. Another limitation of the current work could lie in our decision to focus on tax delinquency instead of other potential indicators of neighborhood instability such as gentrification, foreclosure rates, or bank lending rates. Although such variables may appropriately index neighborhood instability, they are generally unable to address the source distinction (i.e., internal vs. external sources of instability) as each of these components of instability are necessarily external. As such, we relied on property tax delinquency because of its ability to be clearly delineated into two variables based on the property owners (i.e., private individuals vs. companies). We thus caution readers that our work does not speak to the robustness of tax delinquency as a proxy measure to overall instability in relation to other indicators. Finally, our use of LASSO-augmented GWR was intended to serve as a data-driven means to explore these data. We caution readers that these models are indeed exploratory, and that confirmatory work must be done in this area.

### Conclusions

The current work provided evidence that external sources of neighborhood residential instability account for more variability in rates of violence than internal sources. Our findings also revealed that there appears to be a boundary effect on the contagion of violence such that violence is more likely to spread in socially disorganized environments. Future research should aim to study the impact of neighborhood instability from other sources to further confirm our overall assertion that external sources of instability may be stronger predictors of violence events in a given region. Similarly, this work provides an empirical roadmap for how community leaders may reduce violence by revisiting housing laws and investing in resident-led revitalization efforts.

## Supporting information

S1 File Supporting information and supplemental analyses. (DOCX)
