# Supplementary material for: Comparing forms of neighborhood instability as predictors of violence in Richmond, VA
Source: PLoS One. 2022 Sep 6;17(9):e0273718. doi: 10.1371/journal.pone.0273718 (PMC9447869; doi:10.1371/journal.pone.0273718)
Supplement: S1 File — (DOCX) [file pone.0273718.s001.docx]

**Supplemental document 1**

**Negative binomial models**

In order to ensure the findings from our OLS regression models were not due to over dispersed nature of our outcome variable we also conducted two negative binomial (NB) regressions. NB model 1 was largely consistent with OLS model 1, as population density, *IRR* = 1.00, *p* < .001, and company tax delinquency, *IRR* = 1.08, *p* < .001, both significantly predicted violence outcomes, Nagelkerke’s R^2^ = 0.43, *AICc* = 1,007.50. Further, as in OLS model 1, the residuals from NB model 1 still demonstrated significant spatial dependence per Moran’s I, *I* = 0.28, *p* < .001. We conducted the same analysis using personal tax delinquency instead of company delinquency. NB model 2 was similarly consistent with OLS model 2, such that population density, *IRR* = 1.00, *p* < .001, and personal tax delinquency, *IRR* = 1.02, *p* < .001, were both significant predictors of violence events, Nagelkerke’s R^2^ = 0.39, *AICc* = 1,014.31. Further, the residuals from NB model 2 also demonstrated significant spatial dependence, *I* = 0.31, *p* < .001.

**SACSAR models**

To further explore our data, we constructed two spatial simultaneous autocorrelation models: one containing company tax delinquency and another containing personal tax delinquency along with our control variables. We used this approach instead of a spatial error or spatially lagged model because our primary analyses revealed spatial dependency among the violence index data and the model residuals. We constructed two separate models and compared them instead of one model with all predictors due to the high correlation between our tax delinquency variables (i.e., *r* = .87, *p* < .001) as there is currently no software package that would allow us to apply the LASSO to these additional models. We used the *sacsarlm* function from the spatialreg package for these analyses.

**SACSAR model 1: Company tax delinquency**

Our first model contained our control variables and company tax delinquency as independent variables and the violence index as the outcome. This model (rho = -0.45, *z* = -2.62, *p* = .009, AIC = 1150.70) revealed a significant, positive impact of company tax delinquency on the number of violence events observed in a given neighborhood. We also found that population density, alcohol outlets, and the density of food stamp usage were also significant, positive predictors of violence events, whereas the remaining main effects were not significant. Global results are displayed in Table S1.

**Table S1. Standardized estimates of variables in SACSAR model 1.**

| **Variable** | ***β*** | ***z*** | ***p*** |
| --- | --- | --- | --- |
| **Population Density** | 0.39 | 4.49 | < .001 |
| **Company Delinquency** | 0.29 | 4.58 | < .001 |
| **Proportion White** | -0.12 | -1.10 | .269 |
| **Median Income** | -0.07 | -0.80 | .426 |
| **Alcohol Outlets** | 0.16 | 2.46 | .014 |
| **Food Stamps** | 0.38 | 4.33 | < .001 |

We deconstructed the spatially lagged component of Supplemental model 1 using the *impacts* function from the spatialreg package. We implemented a sparse spatial matrix and a 1,000 sample resampling procedure to retrieve estimated test statistics (i.e., z and p-values). Predictor impacts and corresponding p-values are displayed in Table S2.

**Table S2. Standardized estimates of direct, indirect, and total impacts of each predictor in SACSAR model 1.**

| **Variables** | **Direct** | | **Indirect** | | **Total** | |
| --- | --- | --- | --- | --- | --- | --- |
|  | *β* | *z* | *β* | *z* | *β* | *z* |
| **Population Density** | 0.31 | 4.50*** | -0.10 | -2.48* | 0.21 | 4.01*** |
| **Company Delinquency** | 0.31 | 4.55*** | -0.10 | -2.61** | 0.20 | 3.81*** |
| **Proportion White** | -0.12 | -1.15 | 0.04 | 1.01 | -0.08 | -1.13 |
| **Median Income** | -0.08 | -0.84 | 0.03 | 0.80 | -0.05 | -0.82 |
| **Alcohol Outlets** | 0.17 | 2.49* | -0.06 | -1.86 | 0.11 | 2.40* |
| **Food Stamps** | 0.39 | 4.49*** | 0.13 | -2.49* | 0.26 | 4.04*** |

*Note.* * *p* < .05, *p* < .01, ****p* < .001.

**SACSAR model 2: Personal tax delinquency**

SACSAR model 2 was identical to SACSAR model 1 except we replaced the company tax delinquency predictor with personal tax delinquency. This model (rho = -0.49, *z* = -2.94, *p* = .003, AIC = 1149.80) revealed a significant, positive impact of private tax delinquency on the number of violence events observed in a given neighborhood. Similar to SACSAR model 1, we also found that population density, alcohol outlets, and the density of food stamp usage were significant, positive predictors of violence events.

**Table S3.** **Standardized estimates of variables in SACSAR model 2.**

| **Variable** | ***β*** | ***z*** | ***p*** |
| --- | --- | --- | --- |
| **Population Density** | 0.29 | 4.33 | < .001 |
| **Personal Delinquency** | 0.28 | 4.65 | < .001 |
| **Proportion White** | -0.17 | -1.58 | .114 |
| **Median Income** | -0.05 | -0.57 | .568 |
| **Alcohol Outlets** | 0.24 | 3.84 | < .001 |
| **Food Stamps** | 0.35 | 3.95 | < .001 |

**Table S4. Standardized estimates of direct, indirect, and total impacts of each predictor in SACSAR model 2.**

| **Variables** | **Direct** | | **Indirect** | | **Total** | |
| --- | --- | --- | --- | --- | --- | --- |
|  | *β* | *z* | *β* | *z* | *β* | *z* |
| **Population Density** | 0.30 | 4.29*** | -0.11 | -2.57 | 0.19 | 3.76*** |
| **Personal Delinquency** | 0.29 | 4.81*** | -0.11 | -2.94 | 0.19 | 3.81*** |
| **Proportion White** | -0.18 | -1.57 | 0.06 | 1.30 | -0.11 | -1.61 |
| **Median Income** | -0.06 | -0.54 | 0.02 | 0.54 | -0.04 | -0.54 |
| **Alcohol Outlets** | 0.25 | 3.81*** | -0.09 | -2.43 | 0.16 | 3.55*** |
| **Food Stamps** | 0.36 | 3.91*** | -0.13 | -2.53 | 0.23 | 3.51*** |

*Note.* * *p* < .05, *p* < .01, ****p* < .001.

**Relative importance analysis**

To add further context to our analyses we also estimated an OLS model containing all predictor variables to conduct a relative importance analysis. We utilized the *calc.relimp* function from the relaimpo package. We used an OLS model instead of a negative binomial approach as there is currently no software solution available for conducting this type of analysis with a negative binomial model in R. Relative importance of each predictor was determined by finding the average standardized beta for each predictor for each level of model complexity. This analysis revealed that company tax delinquency demonstrated greater importance as a predictor of violence events than private delinquency at every level of model complexity (Table S5).

**Table S5. Average standardized beta estimates of each predictor at varying levels of model complexity.**

|  | **1** | **2** | **3** | **4** | **5** | **6** | **7** |
| --- | --- | --- | --- | --- | --- | --- | --- |
| **Population Density** | 0.43 | 0.37 | 0.34 | 0.31 | 0.28 | 0.26 | 0.25 |
| **Company Delinquency** | 0.45 | 0.38 | 0.33 | 0.28 | 0.24 | 0.20 | 0.16 |
| **Personal Delinquency** | 0.43 | 0.35 | 0.28 | 0.23 | 0.20 | 0.17 | 0.15 |
| **Proportion White** | -0.32 | -0.22 | -0.15 | -0.10 | -0.07 | -0.04 | -0.03 |
| **Median Income** | -0.36 | -0.27 | -0.21 | -0.16 | -0.12 | -0.08 | -0.06 |
| **Alcohol Outlets** | 0.20 | 0.21 | 0.22 | 0.21 | 0.21 | 0.20 | 0.20 |
| **Food Stamps** | 0.45 | 0.40 | 0.37 | 0.36 | 0.34 | 0.33 | 0.32 |

*Note.* Numbers across the top row reflect the number of predictors in the model (i.e., model complexity).
